# Comprehensive multiomics analysis reveals distinct differences between pediatric choroid plexus papilloma and carcinoma

**DOI:** 10.1186/s40478-024-01814-y

**Published:** 2024-06-12

**Authors:** Yeonsong Choi, Seung Ah Choi, Eun Jung Koh, Ilsun Yun, Suhyun Park, Sungwon Jeon, Yeonkyung Kim, Sangbeen Park, Donggeon Woo, Ji Hoon Phi, Sung-Hye Park, Dong-Seok Kim, Se Hoon Kim, Jung Won Choi, Ji Won Lee, Tae-Young Jung, Jong Bhak, Semin Lee, Seung-Ki Kim

**Affiliations:** 1 Department of Biomedical Engineering, College of Information and Biotechnology, Ulsan National Institute of Science and Technology (UNIST), Ulsan, Republic of Korea; 2 Korean Genomics Center, UNIST, Ulsan, Republic of Korea; 3 Division of Pediatric Neurosurgery, Seoul National University Children’s Hospital, Seoul, Republic of Korea; 4 Department of Neurosurgery, Seoul National University Hospital, Seoul National University College of Medicine, Seoul, Republic of Korea; 5 Biomedical Research Institute, Seoul National University Hospital, Seoul, Republic of Korea; 6 Clinomics Inc., Ulsan, Republic of Korea; 7 Neuroscience Research Institute, Seoul National University Medical Research Center, Seoul National University College of Medicine, Seoul, Republic of Korea; 8 Department of Pathology, Seoul National University Hospital, Seoul National University College of Medicine, Seoul, Republic of Korea; 9 Department of Pediatric Neurosurgery, Severance Children’s Hospital, Yonsei University College of Medicine, Seoul, Republic of Korea; 10 Department of Pathology, Severance Hospital, Yonsei University College of Medicine, Seoul, Republic of Korea; 11 Department of Neurosurgery, Samsung Medical Center, Sungkyunkwan University School of Medicine, Seoul, Republic of Korea; 12 Department of Pediatrics, Samsung Medical Center, Sungkyunkwan University School of Medicine, Seoul, Republic of Korea; 13 Department of Neurosurgery, Chonnam National University Medical School and Hwasun Hospital, Hwasun, Republic of Korea

**Keywords:** Choroid plexus tumor, Whole-genome sequencing, Whole-transcriptome sequencing, Methylation sequencing, Multiomics

## Abstract

Choroid plexus tumors (CPTs) are intraventricular tumors derived from the choroid plexus epithelium and occur frequently in children. The aim of this study was to investigate the genomic and epigenomic characteristics of CPT and identify the differences between choroid plexus papilloma (CPP) and choroid plexus carcinoma (CPC). We conducted multiomics analyses of 20 CPT patients including CPP and CPC. Multiomics analysis included whole-genome sequencing, whole-transcriptome sequencing, and methylation sequencing. Mutually exclusive *TP53* and *EPHA7* point mutations, coupled with the amplification of chromosome 1, were exclusively identified in CPC. In contrast, amplification of chromosome 9 was specific to CPP. Differential gene expression analysis uncovered a significant overexpression of genes related to cell cycle regulation and epithelial-mesenchymal transition pathways in CPC compared to CPP. Overexpression of genes associated with tumor metastasis and progression was observed in the CPC subgroup with leptomeningeal dissemination. Furthermore, methylation profiling unveiled hypomethylation in major repeat regions, including long interspersed nuclear elements, short interspersed nuclear elements, long terminal repeats, and retrotransposons in CPC compared to CPP, implying that the loss of epigenetic silencing of transposable elements may play a role in tumorigenesis of CPC. Finally, the differential expression of *AK1*, regulated by both genomic and epigenomic factors, emerged as a potential contributing factor to the histological difference of CPP against CPC. Our results suggest pronounced genomic and epigenomic disparities between CPP and CPC, providing insights into the pathogenesis of CPT at the molecular level.

**Supplementary Information:**

The online version contains supplementary material available at 10.1186/s40478-024-01814-y.

## Introduction

Choroid plexus tumor (CPT) is an intraventricular neoplasm derived from the choroid plexus epithelium [32]. CPT patients are typically diagnosed under the age of five years and account for 2–6% of all pediatric brain tumor patients [38]. The 2021 World Health Organization Classification of Tumors of the Central Nervous System categorizes CPT into three subtypes based on their histological characteristics: choroid plexus papilloma (CPP), atypical choroid plexus papilloma (aCPP) and choroid plexus carcinoma (CPC). CPP shows benign characteristics and is associated with good survival rates, whereas CPC exhibits malignancy, rapid progression, and recurrence [32].

Germline and somatic mutations in *TP53*, a well-known tumor suppressor gene, are among the most common genetic markers of CPC [31]. Moreover, mutations in *TP53* are used to diagnose various diseases, such as Li-Fraumeni syndrome (LFS), which is a cancer predisposition syndrome commonly associated with CPC. [3, 16]. Mutations in *TP53* have been previously reported in 44–67% of CPC patients [31, 36, 52, 56] in studies using targeted sequencing approaches such as Sanger sequencing, panel sequencing, or whole-exome sequencing.

According to previous research, CPT harbors multiple copy number alterations [36, 45, 56], and pediatric CPC frequently shows copy number gains of chromosomes 1, 2, and 21q [56]. In addition, chromosome 9p gain and 10q loss were reported to be associated with longer survival in CPC [45]. Recently, Tong et al. identified *TAF12*, *NFYC*, and *RAD54L*, located on chromosome 1, as oncogenes affecting susceptibility to CPC [57].

Several studies examining the epigenomic aspects of CPT classified benign CPP and malignant CPC according to differential CpG methylation profiles [36, 43, 55]. In particular, Pienkowska et al. reported that *AK1*, *PER2*, and *PLSCR4* were differentially methylated and differentially expressed between CPP and CPC [43].

However, most of the previous studies used single-omics methodologies, which pose limitations in comprehensively characterizing CPT. Although Thomas et al. performed multiomics profiling of CPT using methylation microarray, whole-exome, and RNA sequencing, they focused on the comparison of pediatric and adult CPTs [56]. In this study, we performed comprehensive multiomics analyses of 20 pediatric CPT patients using next-generation sequencing (NGS) techniques encompassing whole-genome sequencing (WGS), whole-transcriptome sequencing (WTS), and methylation sequencing (Methyl-seq) to better understand the genomic and epigenomic characteristics of CPT, focusing on the differences between CPP and CPC.

## Materials and methods

### Patients and samples

To gain genomic and epigenomic insights into CPT, a total of 20 CPT samples were obtained (Table 1). The primary tumors were pathologically diagnosed as CPT, including 6 CPPs, 2 aCPPs, 1 mixed CPP and papillary ependymoma (mCPP), and 11 CPCs. Normal DNA from peripheral white blood cells was available for 11 patients. The patient cohort included 11 females and 9 males, and the average age of the patients was 5.2 years. Thirteen patients had lateral ventricle tumors. Five patients developed lesions of the fourth ventricle. In two cases, the tumors were in the third ventricle. Regarding leptomeningeal seeding (LMS) status, there was no LMS in patients with CPP. However, LMS was found in 5 of the 11 patients with CPC: three patients were diagnosed with LMS preoperatively and LMS was confirmed on spine MRI one month after surgery. All patients with LMS died except for one patient living abroad whose survival could not be confirmed after follow-up.

**Table 1:** Patient information

Sample_ID	Age	Sex	Histology	Location	LMS	Survival	Overall survival (month)	Matched normal	TP53	EPHA7	RT-qPCR (tissues) F4/SF3	RT-qPCR (cells) F4/SF3
SNUH CPP 1	0 (8 m)	M	CPP	3V	−	Alive	99	N			√/√	
SNUH CPP 2	2	M	CPP	LV	−	Alive	83	Y			√/√	
SNUH CPP 3	2	M	CPP	4V	−	Alive	60	N			√/√	√/√
SNUH CPP 4	5	F	CPP	LV	−	Alive	55	N			√/√	
SNUH CPP 5	5	F	CPP	4V	−	Alive	116	N			√/√	
SNUH CPP 6	15	F	CPP	4V	−	Alive	52	Y			√/√	
SNUH aCPP 1	0 (9 m)	F	aCPP	LV	−	Alive	60	Y			√/√	√/√
SNUH aCPP 2	16	F	aCPP	LV	−	Alive	133	N			√/−	
SNUH mCPP 1	5	F	mCPP	LV	−	Alive	86	N			√/−	
SNUH CPC 1	0 (4 m)	M	CPC	LV	−	Alive	5	Y	G		√/√	√/√
SNUH CPC 2	0 (10 m)	M	CPC	LV	+	Dead	7	Y	G		−/√	√/√
SNUH CPC 3	1	F	CPC	3V	+	Dead	14	Y	G		√/√	√/√
SNUH CPC 4	1	M	CPC	4V	+	Dead	9	Y	G		√/√	√/−
SNUH CPC 5	1	F	CPC	LV	−	Alive	137	N		G	√/√	
SNUH CPC 6	2	F	CPC	LV	+	Unknown	16	N	G		√/√	
SNUH CPC 7	5	M	CPC	LV	−	Alive	21	Y	G		√/√	
SNUH CPC 8	9	M	CPC	4V	−	Alive	7	Y	S		√/√	√/−
SNUH CPC 9	9	F	CPC	LV	+	Dead	3	N		G	√/√	
SNUH CPC 10	12	F	CPC	LV	−	Alive	34	Y	G		√/√	
SNUH CPC 11	14	M	CPC	LV	−	Alive	87	Y	G		√/−	

CPP: choroid plexus papilloma, aCPP: atypical choroid plexus papilloma, mCPP: mixed choroid plexus papilloma and papillary ependymoma, CPC: choroid plexus carcinoma, M: male, F: female, LV: lateral ventricle, 3V: third ventricle, 4V: fourth ventricle, LMS: leptomeningeal seeding, overall survival (round to one decimal place), G: (real/predicted) germline mutation, S: (real/predicted) somatic mutation, F4: Fig. 4, SF3: Supplementary Fig. 3

Clinical data were abstracted from medical records. Human specimens were obtained from patients undergoing surgical resection with informed consent for their usage for research purposes in accordance with the guidelines of the Institutional Review Board of Seoul National University Hospital, which approved this study (IRB No. 1507-047-687).

For DNA extraction from samples, a Qiagen QIAamp DNA kit (Qiagen, Valencia, CA) was used. For RNA isolation, a RNeasy Plus Mini Kit (Qiagen) was employed. The concentration and purity of the DNA and RNA were assessed using a spectrophotometer (Denovix Inc., Wilmington, DE). The quality of the purified RNA was validated with a Nanodrop 2000 Spectrophotometer and an Agilent 2100 Bioanalyzer (Agilent Technologies, Santa Clara, CA).

### Cell cultures

Cells were isolated from fresh CPT tissues (2 CPP and 3 CPC) within 4 h of collection. Briefly, the tissues were minced with a surgical knife and dispersed into small aggregates. After enzymatic dissociation into single cells, the cells were filtered and the subsequent processing was performed as described previously [6, 41]. Tumor cells were incubated in Dulbecco's Modified Eagle's Medium (Invitrogen, Carlsbad, CA) supplemented with 10% fetal bovine serum Opti-Gold (GenDEPOT, Katy, TX) and penicillin–streptomycin (× 1 final concentration; Invitrogen). All cells were maintained at 37 °C with 5% CO_2_ in a humidified atmosphere, and only early-passage (p4) cells were used for the validation test.

### Whole-genome sequencing

WGS data were generated from 20 tumor samples and 11 available matched blood samples. WGS libraries were constructed with an input of 0.1–0.5 μg of fragmented DNA using the TruSeq Nano DNA kit (Illumina, Inc., San Diego, CA) following the manufacturer's protocol. The libraries were subjected to an Agilent 2100 Bioanalyzer to estimate the quality and were loaded onto the Illumina NovaSeq 6000 (Theragen Bio, Seongnam, Korea) according to the manufacturer’s recommendations.

Sequencing reads were mapped to the human reference genome (version GRCh38) using Burrows-Wheeler Alignment tool (version 0.7.15) with the “-M” option [28]. Mapped BAM files were sorted and indexed using SAMtools (version 1.9) [7]. Duplicate reads were removed using Picard (version 2.9.0) with the MarkDuplicates module (http://broadinstitute.github.io/picard/). Mapping quality was recalibrated using the BaseRecalibrator and ApplyBQSR tools of the Genome Analysis Toolkit (GATK) (version 4.1.7.0) [34].

Germline single nucleotide variations (SNVs) and short insertions and deletions (INDELs) were called using HaplotypeCaller in GATK (version 4.1.0.0) with the “-ERC GVCF” option and jointly genotyped using the GenotypeGVCFs tool in GATK [34]. The called variants were separated into SNVs and INDELs and were recalibrated using the VariantRecalibrator and ApplyRecalibration tools. Only variants labeled as PASS were used for further analysis and were annotated with a custom pipeline based on ensemble-vep (version 108) [35]. Somatic SNVs and INDELs were called using Mutect2 in GATK (version 4.1.0.0) [34] with the “-af-of-alleles-not-in-resource 0.00003125” and “-max-mnp-distance 0” options and panel of normal constructed 11 normal samples. Only variants labeled as PASS by the “FilterMutectCalls” option were used for further analysis. The remaining variants were annotated with a custom pipeline based on ensemble-vep (version 108) [35]. For somatic mutation analysis, samples with matched normal were detected by paired mode and tumor-only mode, and samples without matched normal were detected by tumor-only mode. Each analysis method was based on the guideline of GATK.

Since matched whole-blood normal samples were not available for some samples, we were limited in distinguishing whether the mutations detected in the tumor are somatic or germline. To account for this, we built a machine learning model to predicted as somatic or germline mutations. After that, mutations with an allele frequency lower than 0.001 in large databases, including the 1000 Genomes Project [14], gnomAD [22], and Korea1K [20] databases, were defined as rare variants.

Somatic copy number alterations (SCNAs) were called by CNVkit (version 0.9.6) with the “-method wgs” and “-target-avg-size 100,000” options for WGS data [53]. Since there were no matched normal data for some patients (9 out of 20 patients), we used a pooled reference that combined all normal samples, as proposed by CNVkit. After utilizing CNVkit, blacklisted regions (centromere regions, with an interval padding of 500,000 bp on each side; telomere regions, 5000 bp on each side) were removed by referring to the University of California Santa Cruz (hg38) blacklist file. Regions with significant focal and arm-level copy number alterations were identified with Genome Identification of Significant Targets in Cancer (GISTIC) [37] using the above copy number alteration profile (*Q* value < 0.05). The oncogene and tumor suppressor gene annotation of significant focal SCNA regions followed the cancer gene consensus (version 90) [50].

### Predicting somatic mutations by machine learning

Predicting somatic point mutations first involves a preprocessed input file constructed with Mutect2 in GATK (version 4.1.0.0) [34] and, allele frequency from the Korea1K database [20]. In addition to these features, we demonstrated the novel feature VAF_z, the *z*-score of the variant allele fraction value between the block ($\pm 1000\text{bp}$) of the mutation.

$$z score(VAF)= \frac{{VAF}_{X}-\mu }{\sigma }$$

$$\left( { \mu = \frac{1}{2000}\mathop \sum \limits_{{X - 1000\,{\text{bp}}}}^{{X + 1000\,{\text{bp}}}} VAF_{i} , \sigma = \sqrt {\frac{1}{2000}\mathop \sum \limits_{{X - 1000\,{\text{bp}}}}^{{X + 1000\,{\text{bp}}}} \left( {VAF_{i} - \mu } \right)^{2} } } \right)$$

Among 102 features as input data, 23 features are defined as important by feature importance evaluation using AutoGluon-Tabular package [9].

Generally, somatic mutations occur rarely compared to germline mutations. Due to this phenomenon, our input data were highly imbalanced. To solve this problem, the WeightedEnsemble L2 model combines 13 classifiers (CatBoost, RandomForestEntr, XGBoost, RandomForestGini, LightGBMLarge, LightGBM, ExtraTreesEntr, ExtraTreesGini, LightGBMXT, NeuralNetTorch, KNeighborsUnif, KNeighborsDist, NeuralNetFastAI). Validation of the model is implemented by fivefold cross validation. We measure the robustness of this model with F1-score and Matthews Correlation Coefficient.

### Clonality analysis

The clonality of the tumor samples was analyzed using FastClone, a state-of-the-art probabilistic tool designed for deconvoluting tumor heterogeneity in bulk-sequencing samples [61]. FastClone is notable for its ability to efficiently and accurately identify subclonal structures by deconvoluting subclones based on its somatic mutations and SCNAs. By modeling the relationships between mutations and the underlying clonal structure with FastClone, we estimated the proportion of cells with a specific set of mutations. Patients without matched blood normal data were excluded since the necessary somatic mutations and SCNAs information could not be accurately provided for clonality analysis.

### Whole-transcriptome sequencing

WTS data were generated from 20 CPT primary tissue samples. WTS libraries were prepared for 151 bp paired-end sequencing using a TruSeq stranded mRNA Sample Preparation Kit (Illumina). mRNA molecules were purified and fragmented from 1 μg of total RNA using oligo (dT) magnetic beads. The fragmented mRNAs were synthesized as single-stranded cDNAs through random hexamer priming. By applying this cDNA as a template for second strand synthesis, double-stranded cDNA was prepared. After a sequential process of end repair, A-tailing and adapter ligation, cDNA libraries were amplified with PCR. The quality of these cDNA libraries was evaluated with the Agilent 2100 BioAnalyzer (Agilent, Santa Clara, CA). The libraries were also quantified with the KAPA library quantification kit (Kapa Biosystems, Wilmington, MA) according to the manufacturer’s library quantification protocol. Following cluster amplification of denatured templates, paired-end sequencing (2 × 151 bp) was performed using an Illumina NovaSeq 6000 (Illumina) instrument.

Gene-level expression values were calculated using a custom pipeline using RSEM (version 1.3.0) [26] with GENCODE (version 33) [12] as a human transcriptome reference (version GRCh38). Differentially expressed gene (DEG) analysis was performed using DESeq2 (version 1.26.0) [33] with the default settings. DEGs with a adjusted *P* value < 0.01 and absolute log_2_(fold change) > 1 were considered significantly up- or downregulated between CPP and CPC, and these genes were used for subsequent analyses. The DEG analysis between the groups with and without LMS was performed equally with DESeq2, genes with − log_10_(padj) > 5 and absolute log_2_ (fold change) > 1 were considered significantly up- or downregulated. principal component analysis (PCA) was performed on the significant DEGs with default parameters. Gene set enrichment analysis was conducted using Enrichr [5, 24, 62], and enriched terms with adjusted *P* values < 0.01 were identified based on the Gene Ontology Biological Process [2, 13], and Molecular Signatures Database [51].

### Methylation sequencing

Methyl-seq data were generated from 20 CPT primary tissue samples. DNA quantification and DNA quality control were performed using Qubit, NanoDrop, and gel electrophoresis. The electrophoresis run was performed on a 0.7% agarose gel for 45 min at 100 V, with 30 ng of DNA loaded. Library preparations were performed according to the SureSelect XT Methyl-Seq Target Enrichment System Kit Protocol (Agilent). Briefly, 3 μg of gDNA was randomly sheared and then DNA fragments of 100–175 bp were extracted. Sheared DNA fragments were end-repaired and purified using AMPure XP beads. The quality of these DNA fragments was evaluated with an Agilent 2100 BioAnalyzer (Agilent). They were quantified with the KAPA library quantification kit (Kapa Biosystems) according to the manufacturer’s library quantification protocol. Following cluster amplification of denatured templates, paired-end sequencing (2 × 151 bp) was performed using Illumina NovaSeq 6000 (Illumina).

Sequencing reads were mapped to the human reference genome (version GRCh38) using Bismark (version 0.18.1) with default options [23]. Mapped BAM files were sorted using SAMtools (version 1.9) [7]. Methylation levels for each CpG site were calculated using MethylKit (version 1.12.0) [1]. To analyze differentially methylated site (DMS), we used calculateDiffMeth in MethylKit and considered CpG sites with a -log *Q* value > 40 and absolute differential methylation value > 55 as significant DMSs. We defined the promoter region as the ~ 1000 bp from the transcription start site using GENCODE (version 33) and calculated the mean beta value of CpG sites within the promoter regions of each gene. PCA was performed on significant DMSs with default settings. GENCODE (version 33), ENCODE Candidate Cis-Regulatory Elements, and RepeatMasker were used to label the CpG sites based on the identity of the genomic regions in which they were located in. The mean beta value of each genomic region was calculated for each sample [10].

### Reverse transcription and quantitative real-time PCR

cDNAs were synthesized from high-quality RNA using the RNA to cDNA EcoDry Premix kit (TAKARA, Shiga, Japan) according to the manufacturer’s instructions. quantitative reverse transcription polymerase chain reaction (RT-qPCR) analysis was carried out by a TaqMan assay on an ABI 7500 system (Applied Biosystems, Foster City, CA) using purchased TaqMan probes (*CDC20*, *LRP2, TMEM265*, *DDTL*, *L1TD1*, and *GABBR1*) and TaqMan® Gene Expression Master Mix (Invitrogen). The relative expression levels in each sample were quantified using the 2-DDCT method. The value of each control sample was set to one and was used to calculate the fold change in target gene expression. The results were normalized to that of GAPDH and were presented relative to the CPP. All data were obtained from three independent experiments, each performed in triplicate.

## Results

### Sequencing statistics

To characterize the genetic and epigenetic variances among the subtypes of CPT, we analyzed 20 CPT patients using WGS, WTS, and Methyl-seq. We produced 31 WGS, 20 WTS, and 20 Methyl-seq data, with average read depths of 43X, 53X, and 131X, respectively. The percentage of sequenced reads mapped to the target region was 99.9%, 97.4%, and 75.6%, respectively for WGS, WTS, and Methyl-seq (Supplementary Table 1).

### Point mutations of *TP53* and *EPHA7* are unique and mutually exclusive in CPC

**Fig. 1:**
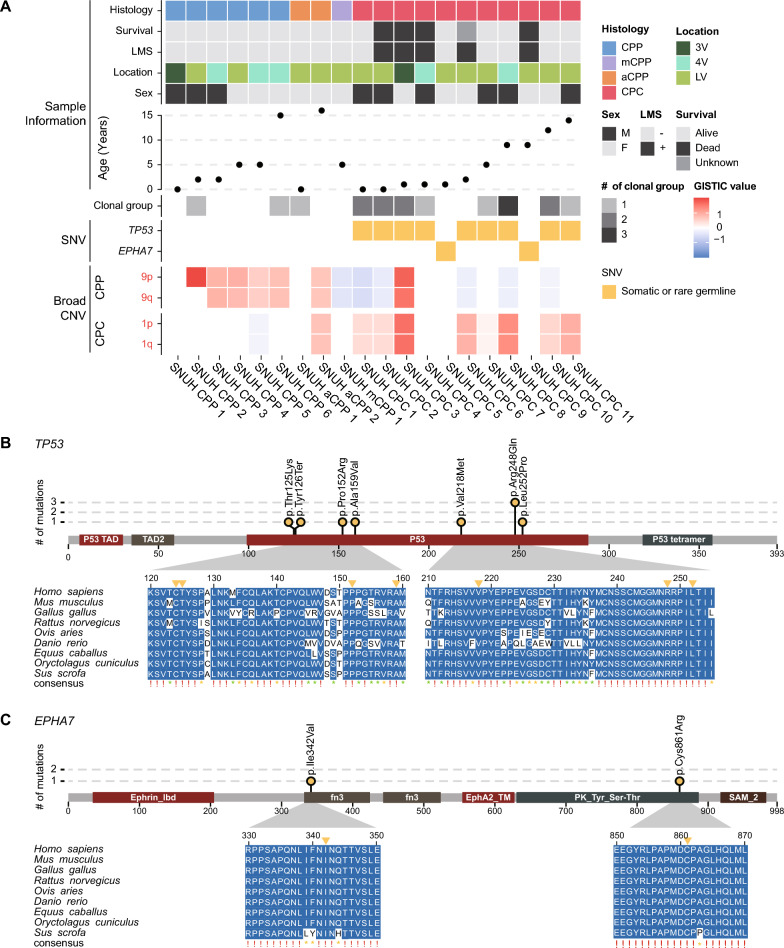
An overview of sample information and genetic events from WGS data. **a** Summary of sample information and genetic events of 20 CPT patients. **b**, **c** Multiple sequence alignment diagrams of regions where point mutations occurred within *TP53* (**b**) and *EPHA7* (**c**), respectively, in several vertebrate species. A red exclamation mark indicates a perfect match across all species examined. A yellow asterisk indicates an 80% or better match across species, but not a perfect match. A green asterisk indicates more than 50% and less than 80% match across species

To identify the genetic variations responsible for CPT, we analyzed point mutations in the WGS data. Notably, rare *TP53* and *EPHA7* variants were discovered only in CPCs (Fig. 1a, Supplementary Table 2). Somatic or rare germline *TP53* mutations occurred in 9 (81.9%) of 11 CPC patients; of these, eight were missense mutations and one was a nonsense mutation. Among them, one *TP53* variant was a somatic mutation that was not detected in the matched blood sample, and the others were rare germline mutations with allele frequencies of less than 0.001 in healthy populations such as Korea1K [20], gnomAD [22], and 1000 Genomes Project [14]. Notably, all these variants were located in the p53 DNA binding domain. Six patients (including one patient with somatic mutation) had variants that were already reported as pathogenic or likely-pathogenic (c.743G > A, c.374C > A, c.652G > A, c.742C > T, and c.476C > T) [11, 25, 29]. Among them, one patient (CPC-1) was diagnosed with LFS with a multiple family history of cancer. Another patient (CPC-2) had a grandmother with breast cancer. Although no family history of cancer was revealed in the medical records of the other four patients, many of these cases might correspond to LFS or Li-Fraumeni-like syndrome. Previous studies have reported a significant association between *TP53* mutations and the development of CPT, particularly CPC [36, 52, 57, 63]. In line with previous studies, *TP53* mutations were observed only in CPC patients in our data. To predict the functional importance of each variant, we performed multiple sequence alignment (MSA). All seven variants occurred at highly conserved positions (Fig. 1b).

Interestingly, CPC patients without *TP53* mutations harbor rare *EPHA7* mutations. *EPHA7* has been identified as a tumor suppressor in various tumors including lymphoma, melanoma, tongue squamous cell carcinoma, and prostate cancer [17, 27, 30, 40, 54]. The two variants identified in this study have not been reported previously. Of the two patients with *EPHA7* mutations, one patient was a long-term survivor, but the other had LMS at the time of diagnosis and died due to rapid progression of the disease. To predict the potential impact associated with the mutations in *EPHA7*, we also performed MSA using the *EPHA7* sequences of several vertebrate species. The sites of the variants in *EPHA7* were highly conserved, implying that the identified mutations in *EPHA7* may impair its function (Fig. 1c).

### Large-scale SCNA reveals distinct differences between CPP and CPC

**Fig. 2:**
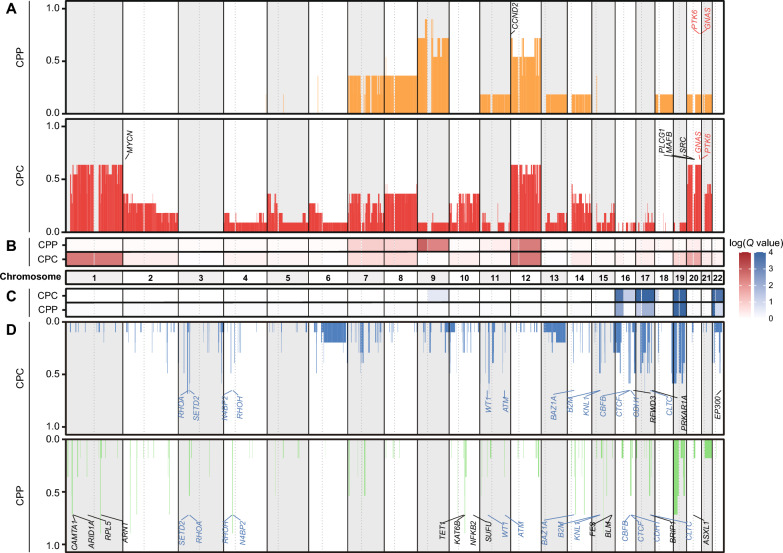
Frequency and significance of SCNAs between CPP and CPC across the autosomal chromosomes. **a** Panel showing the frequency of copy number gain in each subtype. **b** The color bar represents the − log *Q* value of each arm calculated from GISTIC. **c**, **d** Same as **b**, **a**, but represents a copy number loss value

To identify SCNAs in CPT, we analyzed the WGS data of CPT patients. Whole chromosome 12 gain was commonly observed in all subtypes. Whole chromosome 9 gain was repeatedly observed almost exclusively in CPP with 66.7% (4/6) of CPP samples and one CPC sample showing only chromosome 9p gain (Fig. 2a, b). Conversely, whole chromosome 1 gain was detected exclusively in CPC, with 72.7% (8/11) of CPC samples, but not in CPP. The observed whole chromosome 12 gain in CPC is concordant with previous research reporting that more than 70% of CPT patients had chromosome 12 gain [45, 56]. CPP-specific chromosome 9 gain and CPC-specific chromosome 1 gain were also confirmed in previous reports [45, 46, 56]. In addition, losses of chromosomes 16p, 17q, 19, and 22p were commonly found in CPP and CPC. There was no CPP-specific arm-level loss event. Chromosomes 17p, and 22q loss were detected specifically in CPC (Fig. 2c and Supplementary Fig. 1A, B).

There were 6 gains and 28 losses of focal SCNA cytobands commonly detected in both CPP and CPC samples (Supplementary Fig. 1C), which led to amplification of 2 known oncogenes (*GNAS* and *PTK6*) and deletion of 13 known tumor suppressor genes (*ATM, B2M, BAZ1A, CBFB, CDH1, CLTC, CTCF, KNL1, N4BP2, RHOA, RHOH, SETD2*, and *WT1*). In addition, 15 gains and 43 losses of focal SCNA cytobands were detected uniquely in CPP samples, including 1 oncogene amplification (*CCND2*) and 12 tumor suppressor gene deletions (*ARID1A, ARNT, ASXL1, BLM, BRIP1, CAMTA1, FES, KAT6B, NFKB2, RPL5, SUFU*, and *TET1*). On the other hand, 15 gains and 12 losses of focal SCNA cytobands were specifically detected in CPC, including four specific oncogene amplifications (*MAFB, MYCN, PLCG1*, and *SRC*) and three tumor suppressor gene deletions (*EP300, PRKAR1A*, and *RFWD3*) (Fig. 2a, d, Supplementary Table 3).

### CPC has higher intratumoral heterogeneity than CPP

To characterize the genetic clonal structure of CPT subtypes, clonality was predicted for 11 samples with matched blood normal samples based on somatic point mutations and SCNAs profiles. All CPP (n = 2) and aCPP (n = 1) patients were predicted to have one clone, but 62.5% (5 out of 8) of CPC patients (n = 8) were predicted to have two or more clones, with an average of 1.75 clusters (Fig. 1a). Moreover, there was a significant difference in the number of clones between CPP/aCPP and CPC (p = 0.019, Supplementary Fig. 2), suggesting more vigorous tumor evolution in CPC than CPP.

### Cell cycle- and epithelial-mesenchymal transition-related genes are overexpressed in CPC

**Fig. 3:**
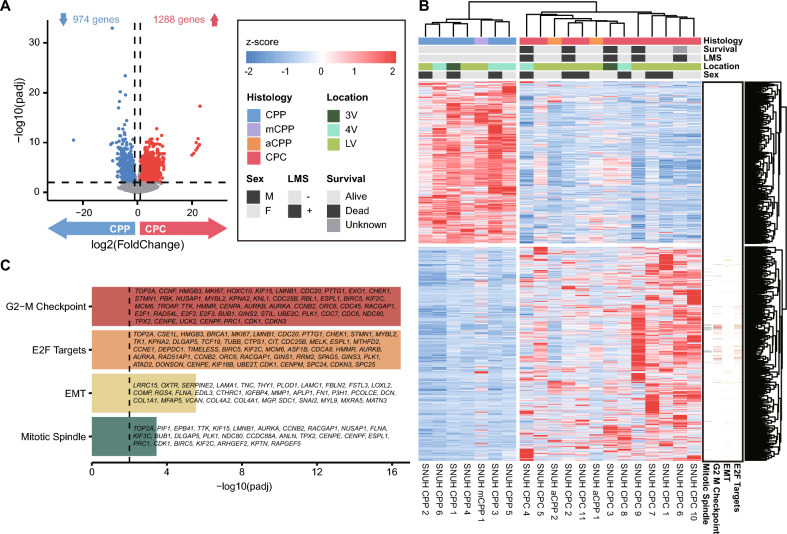
Expression profile of DEGs between the subtypes of CPT. **a** Volcano plot showing genes that are significantly up- and downregulated between CPP and CPC. **b** Heatmap of DEGs between CPP and CPC. Hierarchical clustering was performed by gene (column) and sample (row) according to expression pattern. The top of the figure is colored according to the pathway to which the gene belongs. **c** Enrichment test of significant DEGs and their associated pathways

To identify genes with different expression levels in CPP and CPC, we performed DEG analysis based on expression data, excluding aCPP and mCPP. There were 2,262 genes that showed a significant difference (adjusted *P* value < 0.01, absolute log_2_ (fold change) > 1) in expression levels between the histological subtypes, of which 1,288 genes were upregulated, and 974 genes were downregulated in CPC (Fig. 3a). We performed a hierarchical clustering analysis based on the expression of these DEGs and confirmed the clustering according to the CPP and CPC subtypes (Fig. 3b). The gene expression profiles of aCPP and mCPP were intermediate between those of CPP and CPC. The expression pattern of aCPP resembled that of the CPCs, while that of mCPP was more similar to that of the CPPs.

Additionally, we performed clustering analysis of the DEGs above using the PCA method, and the resulting clusters showed distinct expression profiles by histological subtype (Supplementary Fig. 3A).

Enrichment analysis of the lists of genes upregulated and downregulated between the two histological subtypes was performed. The gene sets significantly upregulated in CPC were associated with the ‘G2-M Checkpoint’, ‘E2F Targets’, ‘Epithelial-Mesenchymal Transition’ and ‘Mitotic Spindle’ pathways (Fig. 3c). The pathway analysis results identified characteristics associated with cell division and migration, indicating the malignant features of CPC. In contrast, the gene set downregulated in CPC was not meaningfully enriched in any pathways.

There were 141 genes that were included in both the significant SCNAs and the DEGs of CPP and CPC. The enrichment analysis of these genes revealed that the ‘G2-M Checkpoint’ pathway was the most significant. Of the 13 genes in this pathway, 10 of them, including *CDC20*, were located on chromosome 1 (Supplementary Table 4). To confirm the expression levels of *CDC20* in CPP and CPC, we performed RT-qPCR. The expression level of *CDC20* was significantly higher in the CPC tissue samples than in the CPP samples. Primary cultured cells could not be tested for statistical significance because the number of samples for CPP is less than 3, but there still seems to be a notable difference in the expression levels (Supplementary Fig. 3B). In a previous study, *RAD54L*, *TAF12*, and *NFYC*, which are co-located on human chromosome 1p, were shown to be involved in the initiation and proliferation of CPC [57]. However, in our data *RAD54L* was the only one of these three genes to satisfy the threshold for classification as a DEG (Supplementary Fig. 3C, Supplementary Table 5).

**Fig. 4:**
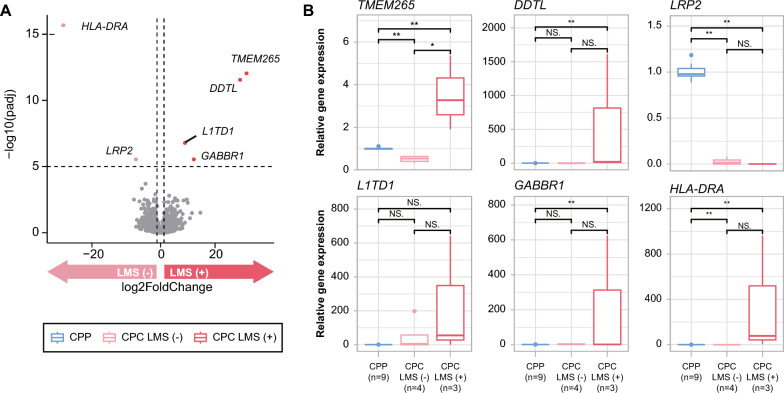
Expression profile of DEGs between LMS (−) and LMS (+) CPC. **a** Volcano plot showing genes that are significantly up- and downregulated depending on LMS status in CPC. **b** PCR validation of DEGs that are significantly up- and downregulated between LMS (−) and LMS (+) CPC. Significant differences between groups are indicated by asterisks. *P < 0.05, **P < 0.01, ***P < 0.001 as calculated by the Wilcoxon rank-sum test

All CPC patients with LMS died, whereas CPC patients without LMS remain alive. LMS was therefore the defining factor in determining the survival outcome of CPC. For this reason, we also performed DEG analysis of CPC patients based on LMS status. Six genes (HLA-*DRA*, *TMEM265*, *DDTL*, *L1TD1*, *GABBR1*, and *LRP2*) showed significantly different expression levels depending on LMS status (-log_10_ (padj) > 5, Fig. 4A). *HLA-DRA* and *LRP2* were downregulated in the group with LMS, while *TMEM265*, *DDTL*, *L1TD1*, and *GABBR1* were upregulated in the group with LMS (Supplementary Table 6). In particular, *L1TD1* and *GABBR1* have been previously associated with tumor metastasis and progression in other cancers. *L1TD1* is highly expressed in seminoma, embryonal carcinoma, and medulloblastoma cancer cells, and its upregulation is associated with poor clinical outcome and metastasis of medulloblastoma. *L1TD1* knockdown results in downregulation of pluripotency factors and reduced proliferation, as well as decreased migration and invasion capacity of medulloblastoma cells [39, 47]. There is accumulating evidence implicating *GABBR1* in the cancer cell growth and metastasis of high-grade chondrosarcoma, breast cancer, and renal cell carcinoma [18, 21, 59]. Furthermore, previous methylation analysis studies of CPT have demonstrated that GABA receptor signaling is the most enriched pathway in CPC [43]. We further validated the differential expression of those genes in tissues through RT-qPCR. Among the six genes, five genes (*LRP2, TMEM265*, *DDTL*, *L1TD1*, and *GABBR1*) showed concordant expression patterns, and *TMEM265* showed a statistically significant difference in its expression levels (Fig. 4b).

### CPC and CPP show differential methylation patterns in repeat regions

**Fig. 5:**
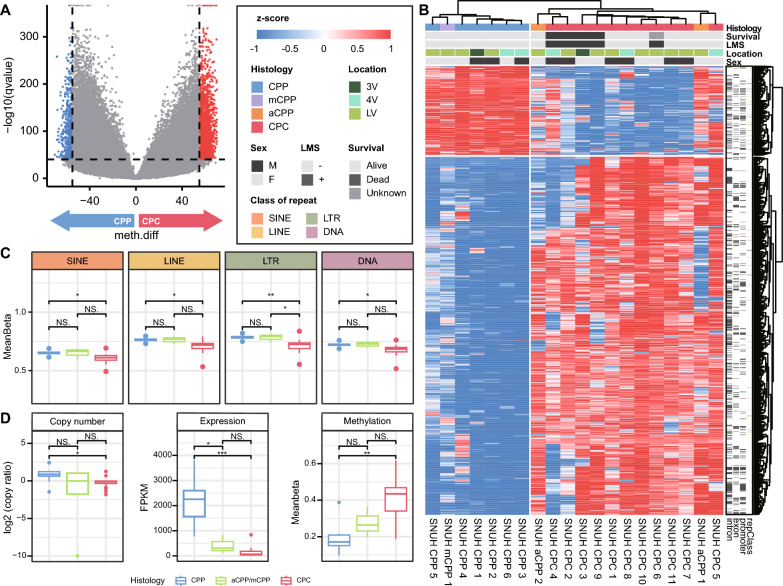
Methylation profile of DMSs between CPT subtypes. **a** Volcano plot showing sites that are significantly hyper- and hypomethylated sites between CPP and CPC. **b** Heatmap of DMSs between CPP and CPC. Hierarchical clustering is performed by CpG site (column) and sample (row) according to the methylation pattern. The top of the figure indicates the genomic regions in which each site is located. **c** Boxplot of the degree of methylation in four types of repeat regions, grouped by CPT subtype. Significant differences between groups are indicated by asterisks.: *P < 0.05, **P < 0.01 as calculated by *t* test. **d** Copy number ratio, expression, and methylation of *AK1* in different CPT subtypes. Significant differences between groups are indicated by asterisks.: *P < 0.05, **P < 0.01, ***P < 0.001 as calculated by the Wilcoxon rank-sum test

To identify distinct methylation patterns in CPP and CPC, we performed a DMS analysis. There were 2,139 significantly different CpG sites (-log *Q* value > 40, absolute differential methylation value > 55), including 1,709 hypermethylation and 430 hypomethylation sites. When clustering was performed with CpG sites with significant methylation differences, two clusters formed, one for each CPT subtype (Fig. 5a, b). In addition, aCPPs were clustered with CPCs, but mCPP was grouped with CPPs. In PCA with the corresponding sites, samples of the same subtype of CPT clustered closely together (Supplementary Fig. 4A).

We examined differences in methylation according to genomic region, including protein-coding regions, cis-regulatory elements, and repeat regions. There was no significant difference in the degree of methylation in the genomic regions except for the repeat regions (Supplementary Fig. 4B). Interestingly, the degree of methylation in major repeat regions such as long interspersed nuclear elements (LINEs), short interspersed nuclear elements (SINEs), long terminal repeats (LTRs), and DNA transposon regions was significantly lower in CPC than in CPP (Fig. 5c).

In the promoter region, we identified 746 hypermethylation sites involving 112 genes and 332 hypomethylation sites involving 83 genes. Notably, we found that the promoter regions of p53-related genes were hypermethylated, while those related to angiogenesis were hypomethylated in CPC. There were only significantly enriched pathways in each methylation status. Specifically, *AK1*, *KIF13B*, *PLXNB2*, and *RALGDS* were found to be involved in the ‘p53 pathway’, whereas *PDGFA* and *OLR1* were involved in ‘angiogenesis’. These results suggest the tumorigenic characteristics of CPC. (Supplementary Fig. 4C).

### The differential expression of AK1 driven by genomic and epigenomic factors in CPP

To identify the genes whose expression was affected by methylation, we compared the gene lists from the DMS analysis and the DEG analysis. As a result, we discovered four overlapping genes (*FABP3, PDE6G, FAM24B*, and *CNN3*) between the hypomethylated and upregulated genes and 16 overlapping genes (*SYNE1*, *APOBEC4*, *SLC20A2*, *GAS2L2*, *AL031710*.1, *AC027449*.1, *AK1*, *AKNA*, *SPAG8*, *RALGDS*, *TCTEX1D4*, *AP000842.2, MIR600HG, DDO, TRIM29*, and *FAM102A*) between the hypermethylated and downregulated genes.

Interestingly, among the genes identified through the DMS pathway analysis, *AK1* showed significant differences in its expression level (Fig. 5d). Previous studies have identified *AK1* as a methylation signature in CPC, and its downregulation is documented not only in CPC but also in various other cancer types [19, 43, 58]. Additionally, recent research on colorectal cancer has revealed an increased expression of the adenylate kinase (AK) genes in benign polyps, followed by a decrease in expression levels of AK genes in cancer [44]. Although CPP does not seem to develop into CPC, the significant difference in expression of *AK1* may cause the differences in histological characteristics between the two CPT subtypes. We observed that the amplification of chromosome 9 resulted in the increased copy number of *AK1* in CPP. Therefore, we hypothesized that both the copy number gain and the hypomethylation of *AK1* in CPP may have driven the overexpression of *AK1*, proposing that *AK1* be one of the candidates driving the difference between CPC and CPP.

## Discussion

The primary objective of this study was to investigate the genomic and epigenomic characteristics of CPT and discern the differences between CPP and CPC. To achieve this, we conducted comprehensive multiomics analyses of 20 pediatric CPT patients, employing NGS techniques, including WGS, WTS, and Methyl-seq.

Our findings revealed distinctive *TP53* alterations in CPC, along with novel *EPHA7* point mutations specifically in TP53-wild-type CPC. Both genes, recognized for their tumor suppressor roles, may play a crucial role in the pathogenesis of CPC. *TP53* mutations in CPC have been reported in several CPT studies [36, 52, 63]. On the other hand, *EPHA7* has not been studied in choroid plexus tumors but has been identified as a tumor suppressor in various other cancers [17, 27, 30, 40, 54]. Our study implies that mutation of *EPHA7* may have resulted in the loss of its tumor suppressor properties and the promotion of carcinoma progression in choroid plexus tumor. However, further research is required to understand the specific mechanism of the anti-tumor effect of wild-type *EPHA7* and the oncogenesis of mutant *EPHA7* in choroid plexus tumors. Due to small numbers of patients with an *EPHA7* mutation, it is difficult to determine the association between the *EPHA7* mutation and the prognosis of CPC. Among the two patients with an *EPHA7* mutation, one patient is a long-term survivor, but the other had LMS at the time of diagnosis and died of rapid progression of disease.

SCNAs at the arm level emerged as characteristic features for each CPT subtype. Chromosome 12 gain was prevalent in CPT patients, while chromosome 9 and chromosome 1 gains were exclusively associated with CPP and CPC, respectively. Our findings suggest that SCNAs play an important role in CPT development and progression. Kaishi Satomi et al., although not analyzing CPT patients, observed that chromosome 12 gain is the most frequently copy number alteration in central nervous system germ cell tumors [48]. CPP-specific chromosomal 9 gain and CPC-specific chromosomal 1 gain were also previously reported [45, 46, 56]. Chromosome 9 gain has been associated with good survival in CPC [45]. However, chromosome 9 gain was mainly not observed in CPC whereas it was identified dominantly in CPP patients in our results. One CPC patient with chromosome 9 gain had LMS and expired due to disease progression.

Furthermore, a significantly higher number of clones were observed in CPC compared to CPP, indicating a higher intratumoral heterogeneity. As far as we know, this is the first analysis to perform clonality analysis of CPT with WGS data. We showed that significantly more clusters were predicted in CPC than in CPP, and these results may reflect the tendency for relapse and therapeutic resistance in CPC.

Notably, overexpression of cell cycle-related genes located on chromosome 1 was identified in CPC, contributing to carcinoma characteristics. According to a previous study, some genes located on chromosome 1p were involved in the initiation and proliferation of CPC, but our study found that some of these genes did not satisfy our thresholds in the DEG analysis between CPP and CPC [57]. Especially, DEG analysis between CPC patients with different LMS status showed that CPC patients with LMS had significantly higher expression levels of genes including *L1TD1* and *GABBR1*, which are known to be associated with tumor migration and invasion in other solid tumors [4, 8, 15, 42, 47, 49, 60, 64]. RT-qPCR experiments confirmed that the expression patterns of DEGs between LMS + and LMS- CPCs were generally concordant with WTS results although only *TMEM265* showed statistical significance. This may be due to the small sample size used in the experiments.

Additionally, to the best of our knowledge, our study is the first to report hypomethylation in various types of repeat regions in CPC, implying the potential influence of the loss of epigenetic silencing of transposable elements on CPC development. We also highlighted the differential expression of *AK1* as a distinguishing factor between CPP and CPC, potentially influenced by both genomic and epigenomic factors. Downregulation of *AK1* has been reported in several cancers [19, 58], and hypermethylation and downregulation of the AK1 promoter region in choroid plexus tumors have already been reported [43]. Our results are consistent with this, and furthermore, our analysis suggests that *AK1* upregulation in CPP compared to CPC is induced by the amplification of chromosome 9 as well as the hypomethylation of *AK1*.

Although aCPP and mCPP have generally intermediate properties based on their gene expression and methylation profiles, a closer look at the clustering results revealed that aCPP was closer to CPC while mCPP was more similar to CPP. However, due to the small sample size, the results should be interpreted with caution.

There are some limitations in this study, including a relatively small sample size due to the rarity of pediatric CPT. Continuous efforts to secure additional samples from multiple centers are crucial. Additionally, there was a lack of age-matched normal choroid plexus tissues and normal blood samples for some patients limited our ability to conduct a comprehensive comparison. To address this, we utilized large-scale healthy population-based variant databases as controls.

## Conclusions

**Fig. 6:**
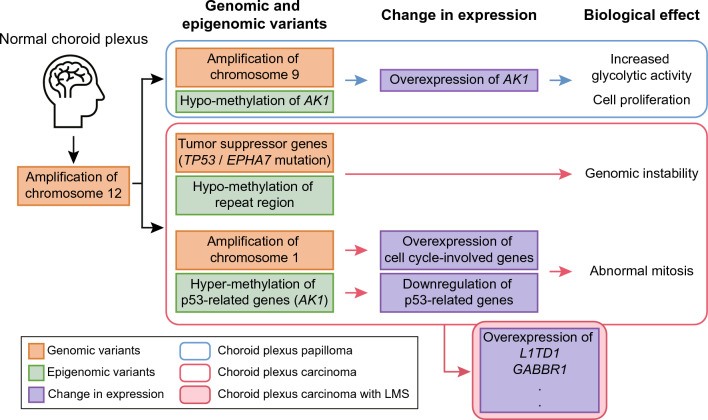
Summary of genomic and epigenomic differences between CPP and CPC

In conclusion, our comprehensive multiomics analysis of 20 pediatric CPT patients using various NGS techniques provides valuable insights into the difference of CPT subtypes. We suggest that the difference between CPP and CPC arise during the initial stages of progression and develop benign and malignant characteristics, respectively (Fig. 6). The identified genomic and epigenomic characteristics could contribute to the understanding of CPT pathogenesis. In addition, this study may provide insight for the development of novel therapeutic strategies aimed at addressing the specific molecular intricacies associated with CPT.

### Supplementary Information

Supplementary Material 1

Supplementary Material 2

Supplementary Material 3

## Data Availability

All the raw data generated and used in this study have been deposited in the Sequence Read Archive (SRA, https://www.ncbi.nlm.nih.gov/sra) under the submission ID, PRJNA1107711 (link for reviewer: https://dataview.ncbi.nlm.nih.gov/object/PRJNA1107711?reviewer=njv4q0jujhbdcrf50sanjikgsj). We have also deposited our processed data at GEO (WTS: https://www.ncbi.nlm.nih.gov/geo/query/acc.cgi?acc=GSE267874, BisulfiteSeq: https://www.ncbi.nlm.nih.gov/geo/query/acc.cgi?acc=GSE267876).

## Abbreviations

CPT: Choroid plexus tumor
CPP: Choroid plexus papilloma
aCPP: Atypical choroid plexus papilloma
mCPP: Mixed CPP and papillary ependymoma
CPC: Choroid plexus carcinoma
LFS: Li-Fraumeni syndrome
NGS: Next-generation sequencing
WGS: Whole-genome sequencing
WTS: Whole-transcriptome sequencing
Methyl-seq: Methylation sequencing
LMS: Leptomeningeal seeding
GATK: Genome analysis toolkit
SNV: Single nucleotide variation
INDEL: Insertion and deletion
SCNA: Somatic copy number alteration
GISTIC: Genome identification of significant targets in cancer
