# Supplementary material for: Comprehensive multiomics analysis reveals distinct differences between pediatric choroid plexus papilloma and carcinoma
Source: Acta Neuropathol Commun. 2024 Jun 12;12:93. doi: 10.1186/s40478-024-01814-y (PMC11167863; doi:10.1186/s40478-024-01814-y)
Supplement: Supplementary file 1 — Supplementary Material 1 [file 40478_2024_1814_MOESM1_ESM.docx]

**Supplementary Table 1. Summary of sequencing statistics**

| Sample ID | Tissue | Average Read Depth | | | % of Aligned Read | | |
| --- | --- | --- | --- | --- | --- | --- | --- |
|  |  | **WGS** | **WTS** | **Methyl-seq** | **WGS** | **WTS** | **Methyl-seq** |
| **SNUH_CPP_1** | **Tumor** | 34.1 | 64.8 | 117.5 | 99.9% | 98.4% | 81.0% |
| **SNUH_CPP_2** | **Tumor** | 77.0 | 69.8 | 124.7 | 99.8% | 98.0% | 78.0% |
| **SNUH_CPP_3** | **Tumor** | 40.2 | 59.0 | 114.3 | 99.8% | 98.1% | 78.6% |
| **SNUH_CPP_4** | **Tumor** | 34.3 | 47.2 | 132.5 | 99.9% | 97.5% | 70.3% |
| **SNUH_CPP_5** | **Tumor** | 40.9 | 59.4 | 134.9 | 99.9% | 97.1% | 82.6% |
| **SNUH_CPP_6** | **Tumor** | 34.5 | 41.4 | 136.9 | 99.8% | 97.3% | 77.5% |
| **SNUH_aCPP_1** | **Tumor** | 33.8 | 62.4 | 148.7 | 99.9% | 98.4% | 79.3% |
| **SNUH_aCPP_2** | **Tumor** | 90.8 | 82.5 | 120.3 | 99.8% | 98.6% | 79.7% |
| **SNUH_mCPP_1** | **Tumor** | 35.3 | 58.7 | 141.0 | 99.9% | 98.5% | 78.8% |
| **SNUH_CPC_1** | **Tumor** | 56.5 | 30.9 | 99.2 | 99.7% | 92.9% | 41.7% |
| **SNUH_CPC_2** | **Tumor** | 34.9 | 58.4 | 147.7 | 99.9% | 98.3% | 80.7% |
| **SNUH_CPC_3** | **Tumor** | 86.5 | 58.4 | 119.9 | 99.8% | 97.5% | 78.3% |
| **SNUH_CPC_4** | **Tumor** | 36.2 | 45.3 | 147.9 | 99.9% | 94.6% | 71.2% |
| **SNUH_CPC_5** | **Tumor** | 33.6 | 77.1 | 137.0 | 99.9% | 98.5% | 79.8% |
| **SNUH_CPC_6** | **Tumor** | 33.9 | 37.5 | 114.6 | 99.9% | 98.2% | 75.0% |
| **SNUH_CPC_7** | **Tumor** | 34.8 | 26.2 | 145.7 | 99.9% | 97.5% | 78.4% |
| **SNUH_CPC_8** | **Tumor** | 54.6 | 24.9 | 120.4 | 99.8% | 93.7% | 63.8% |
| **SNUH_CPC_9** | **Tumor** | 41.6 | 29.4 | 137.3 | 99.9% | 97.4% | 79.6% |
| **SNUH_CPC_10** | **Tumor** | 43.3 | 50.6 | 134.6 | 99.8% | 98.4% | 79.7% |
| **SNUH_CPC_11** | **Tumor** | 42.2 | 75.4 | 146.4 | 99.8% | 98.7% | 77.9% |
| **SNUH_CPP_2** | **Blood** | 36.6 | NA | NA | 99.9% | NA | NA |
| **SNUH_CPP_6** | **Blood** | 43.1 | NA | NA | 99.9% | NA | NA |
| **SNUH_aCPP_1** | **Blood** | 36.8 | NA | NA | 99.9% | NA | NA |
| **SNUH_CPC_1** | **Blood** | 26.4 | NA | NA | 99.7% | NA | NA |
| **SNUH_CPC_2** | **Blood** | 41.4 | NA | NA | 99.9% | NA | NA |
| **SNUH_CPC_3** | **Blood** | 40.7 | NA | NA | 99.9% | NA | NA |
| **SNUH_CPC_4** | **Blood** | 41.8 | NA | NA | 99.8% | NA | NA |
| **SNUH_CPC_7** | **Blood** | 34.9 | NA | NA | 99.9% | NA | NA |
| **SNUH_CPC_8** | **Blood** | 28.4 | NA | NA | 99.7% | NA | NA |
| **SNUH_CPC_10** | **Blood** | 36.9 | NA | NA | 99.9% | NA | NA |
| **SNUH_CPC_11** | **Blood** | 36.6 | NA | NA | 99.9% | NA | NA |

**Supplementary Table 2. Summary of point mutations**

|  | Gene | Chr | Position | Variant Classification | Reference allele | Tumor allele | Sample ID | HGVSc | HGVSp | Genomic Identifiers | dbSNP |
| --- | --- | --- | --- | --- | --- | --- | --- | --- | --- | --- | --- |
| *1* | *TP53* | chr17 | 7674220 | Missense | C | T | SNUH CPC 1 | c.743G>A | p.Arg248Gln | COSV52661580 | rs11540652 |
| *2* | *TP53* | chr17 | 7675995 | Missense | G | T | SNUH CPC 4 | c.374C>A | p.Thr125Lys | COSV52667346 | rs786201057 |
| *3* | *TP53* | chr17 | 7674879 | Missense | C | T | SNUH CPC 11 | c.652G>A | p.Val218Met | COSV52795822 | rs878854072 |
| *4* | *TP53* | chr17 | 7674221 | Missense | G | A | SNUH CPC 2 | c.742C>T | p.Arg248Trp | COSV52662035 | rs121912651 |
| *5* | *TP53* | chr17 | 7675136 | Missense | G | A | SNUH CPC 3 | c.476C>T | p.Ala159Val | COSV52674926 | rs1555526131 |
| *6* | *TP53* | chr17 | 7675234 | Nonsense | G | C | SNUH CPC 10 | c.378C>G | p.Tyr126Ter | COSV52662583 | rs1567554500 |
| *7* | *TP53* | chr17 | 7675157 | Missense | G | C | SNUH CPC 7 | c.455C>G | p.Pro152Arg | COSV52773466 | rs587782705 |
| *8* | *TP53* | chr17 | 7674208 | Missense | A | G | SNUH CPC 6 | c.755T>C | p.Leu252Pro | COSV52764738 | rs121912653 |
| *9* | *TP53* | chr17 | 7674220 | Missense | C | T | SNUH CPC 8 | c.743G>A | p.Arg248Gln | COSV52661580 | rs11540652 |
| *10* | *EPHA7* | chr6 | 93357017 | Missense | T | C | SNUH CPC 9 | c.1024A>G | p.Ile342Val | NA | NA |
| *11* | *EPHA7* | chr6 | 93246937 | Missense | A | G | SNUH CPC 5 | c.2581T>C | p.Cys861Arg | NA | rs1393547166 |

|  | SIFT | PolyPhen | ClinVar | AF | EAS AF | gnomAD AF | gnomAD EAS AF | Korea1K AF | Somatic/  Germline |
| --- | --- | --- | --- | --- | --- | --- | --- | --- | --- |
| *1* | deleterious(0.03) | probably_damaging(0.994) | pathogenic&pathogenic/likely_pathogenic&likely_pathogenic | 0 | 0 | 2.03E-05 | 5.80E-05 | 0 | Germline |
| *2* | deleterious(0) | probably_damaging(0.999) | uncertain_significance&likely_pathogenic | 0 | 0 | 0 | 0 | 0 | Germline |
| *3* | deleterious(0.02) | probably_damaging(0.997) | pathogenic | 0 | 0 | 0 | 0 | 0 | Germline |
| *4* | deleterious(0) | probably_damaging(1) | pathogenic&likely_pathogenic | 0 | 0 | 4.06E-06 | 0 | 0 | Germline |
| *5* | tolerated(0.09) | possibly_damaging(0.552) | uncertain_significance | 0 | 0 | 0 | 0 | 0 | Germline |
| *6* | NA | NA | likely_pathogenic | 0 | 0 | 0 | 0 | 0 | Germline |
| *7* | deleterious(0) | probably_damaging(0.997) | NA | 0 | 0 | 0 | 0 | 0 | Germline |
| *8* | deleterious(0.01) | probably_damaging(0.987) | pathogenic | 0 | 0 | 0 | 0 | 0 | Germline |
| *9* | deleterious(0.03) | probably_damaging(0.994) | pathogenic&pathogenic/likely_pathogenic&likely_pathogenic | 0 | 0 | 2.03E-05 | 5.80E-05 | 0 | Somatic |
| *10* | tolerated(1) | benign(0.05) | NA | 0 | 0 | 0 | 0 | 0 | Germline |
| *11* | deleterious(0) | probably_damaging(1) | NA | 0 | 0 | 0 | 0 | 0 | Germline |

AF: frequency of existing variant in 1000 Genomes; EAS AF: frequency of existing variant in 1000 Genomes combined East Asian population

**Supplementary Table 3. Focal SCNAs in CPT subtypes**

|  | Gain | | Loss | |
| --- | --- | --- | --- | --- |
|  | Cytoband | Oncogene | Cytoband | Tumor suppressor gene |
| Common | 6 | 2 | 28 | 13 |
| CPP | 15 | 1 | 43 | 12 |
| CPC | 15 | 4 | 12 | 3 |

**Supplementary Table 4. Enrichment analysis of overlapping genes between significant DEGs and SCNAs**

| **Term** | **Overlap** | **P-value** | **Adjusted P-value** | **Odds Ratio** | **Combined Score** | **Genes** |
| --- | --- | --- | --- | --- | --- | --- |
| G2-M Checkpoint | 13/200 | 1.49E-06 | 5.05E-05 | 5.74 | 77.01 | *STIL, UBE2C, CDC7, AURKA, CDC20, UCK2, CENPF, EXO1, STMN1, RAD54L, E2F2, KIF2C, MYBL2* |
| E2F Targets | 10/200 | 2.20E-04 | 0.003732 | 4.29 | 36.13 | *CDC20, CSE1L, UBE2T, STMN1, DEPDC1, CDCA8, KIF2C, MYBL2, CTPS1, AURKA* |

**Supplementary Table 5. DEG analysis of previously reported choroid plexus carcinoma oncogenes in Tong et al[54].**

| ID | baseMean | log2FoldChange | lfcSE | stat | pvalue | padj |
| --- | --- | --- | --- | --- | --- | --- |
| *RAD54L* | 215.7381 | 3.418818 | 0.586662 | 5.827578 | 5.62E-09 | 7.88E-07 |
| *TAF12* | 999.6316 | 1.011748 | 0.341952 | 2.958743 | 0.003089 | 0.027222 |
| *NFYC* | 3073.608 | 0.25111 | 0.252894 | 0.992947 | 0.320736 | 0.578958 |

**Supplementary Table 6. DEGs between LMS (-) and LMS (+) CPC**

| SYMBOL | p_adj_ | DGE | Reference |
| --- | --- | --- | --- |
| *TMEM265* | 9.14E-13 | UP | Exploration of a Novel Prognostic Risk Signature and Its Effect on the Immune Response in Nasopharyngeal Carcinoma |
| *DDTL* | 2.80E-12 | UP | Screen and classify genes on bladder cancer associated with metastasis |
| *L1TD1* | 1.63E-07 | UP | L1TD1 - a prognostic marker for colon cancer |
|  |  |  | Embryonic Stem Cell-Related Protein L1TD1 Is Required for Cell Viability, Neurosphere Formation, and Chemoresistance in Medulloblastoma |
| *GABBR1* | 2.86E-06 | UP | GABBR1 as a predictor of prognosis and Its correlation with immune infiltration and dysfunction in clear cell renal cell carcinoma |
| *HLA-DRA* | 2.17E-16 | DOWN | HLA-DR expression in tumor epithelium is an independent prognostic indicator in esophageal adenocarcinoma patients |
|  |  |  | A prognostic immune predictor, HLA-DRA, plays diverse roles in non-muscle invasive and muscle invasive bladder cancer |
| *LRP2* | 2.86E-06 | DOWN | Expression of LDL receptor-related proteins (LRPs) in common solid malignancies correlates with patient survival |
