# Supplementary material for: Comprehensive multiomics analysis reveals distinct differences between pediatric choroid plexus papilloma and carcinoma
Source: Acta Neuropathol Commun. 2024 Jun 12;12:93. doi: 10.1186/s40478-024-01814-y (PMC11167863; doi:10.1186/s40478-024-01814-y)

Supplementary Figure 1

A

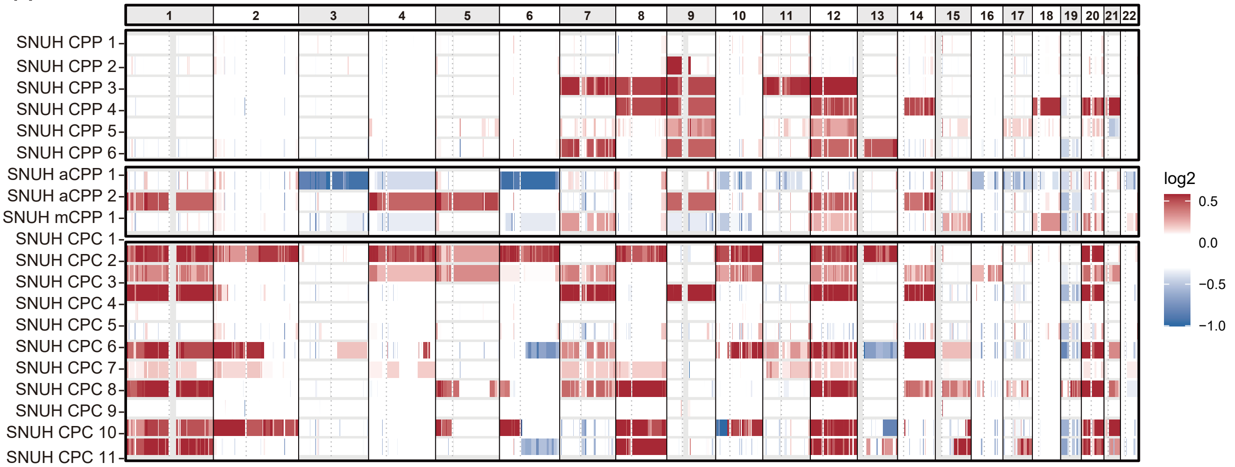

B

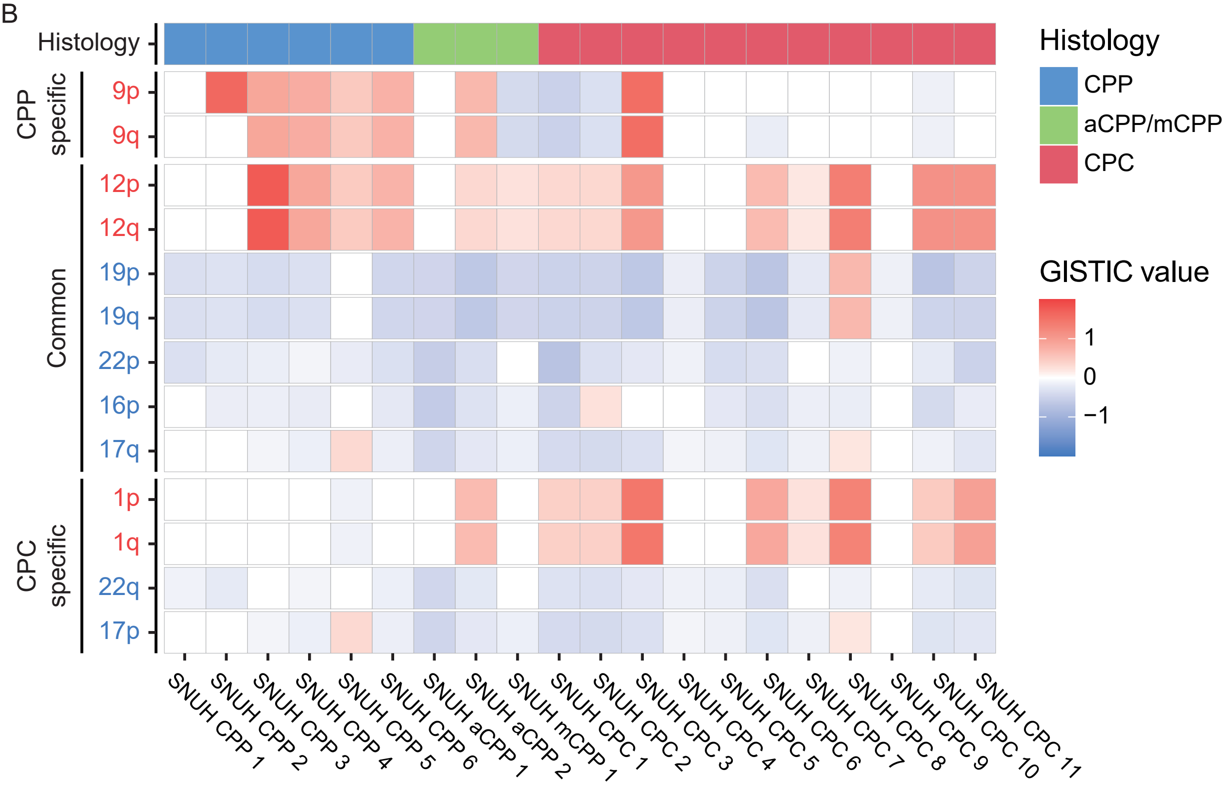

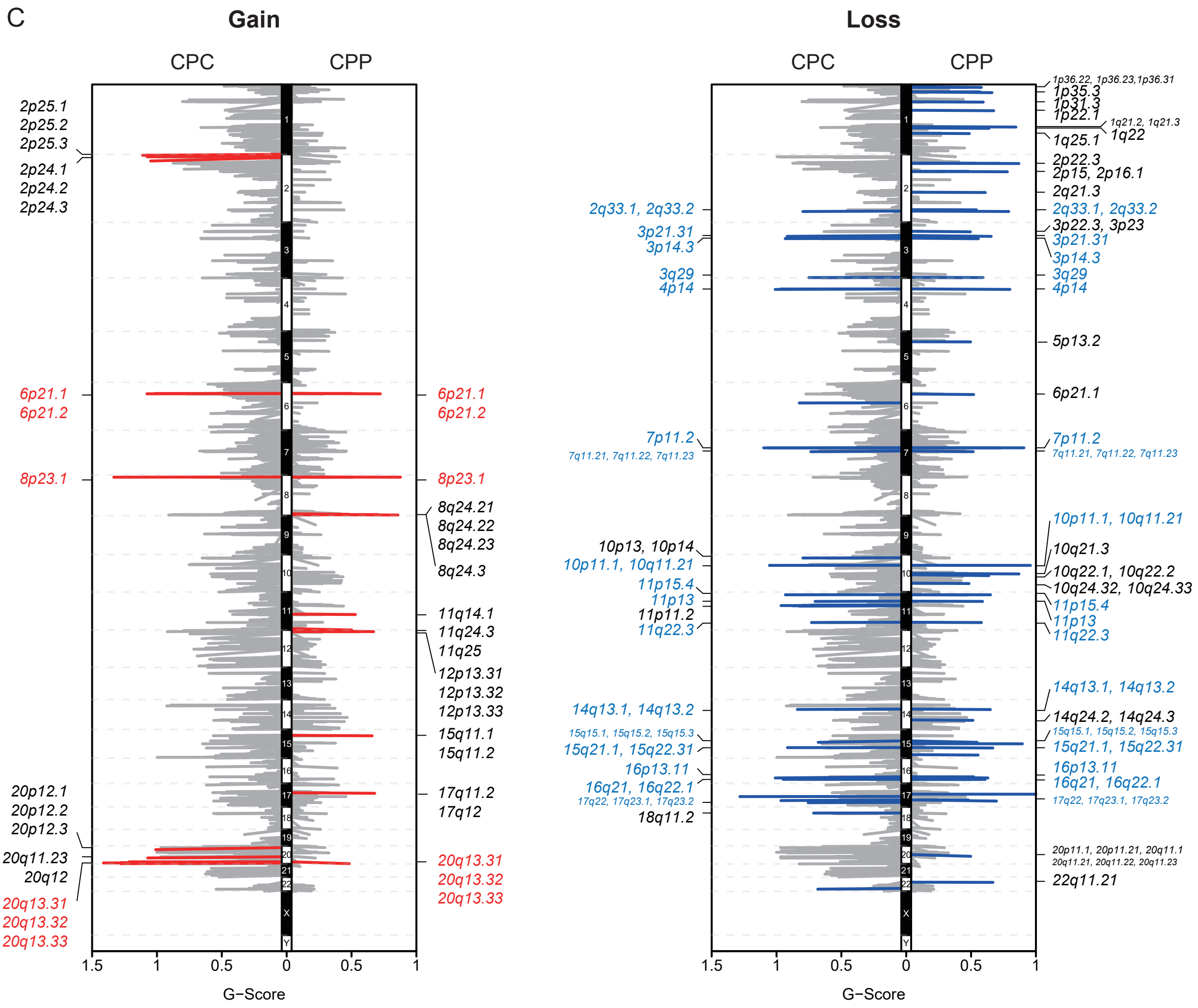
C
Gain
Loss
CPC
CPP
2p25.1
2p25.2
2p25.3
2p24.1
2p24.2
2p24.3
6p21.1
6p21.2
8p23.1
8q24.21
8q24.22
8q24.23
8q24.3
11q14.1
11q24.3
11q25
12p13.31
12p13.32
12p13.33
15q11.1
15q11.2
17q11.2
17q12
20p12.1
20p12.2
20p12.3
20q11.23
20q12
20q13.31
20q13.32
20q13.33
1p36.22, 1p36.23,1p36.31
1p35.3
1p31.3
1p22.1
1q21.2, 1q21.3
1q22
1q25.1
2p22.3
2p15, 2p16.1
2q21.3
2q33.1, 2q33.2
3p22.3, 3p23
3p21.31
3p14.3
3q29
4p14
5p13.2
6p21.1
7p11.2
7q11.21, 7q11.22, 7q11.23
10p11.1, 10q11.21
10q21.3
10q22.1, 10q22.2
10q24.32, 10q24.33
10p13, 10p14
11p15.4
11p13
11p11.2
11q22.3
14q13.1, 14q13.2
14q24.2, 14q24.3
15q15.1, 15q15.2, 15q15.3
15q21.1, 15q22.31
16p13.11
16q21, 16q22.1
17q22, 17q23.1, 17q23.2
18q11.2
20p11.1, 20p11.21, 20q11.1
20q11.21, 20q11.22, 20q11.23
22q11.21
G−Score
1.5
1
0.5
0

Supplementary Figure 2

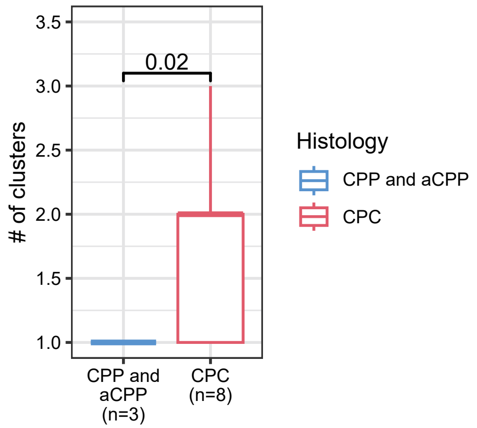

Supplementary Figure 3

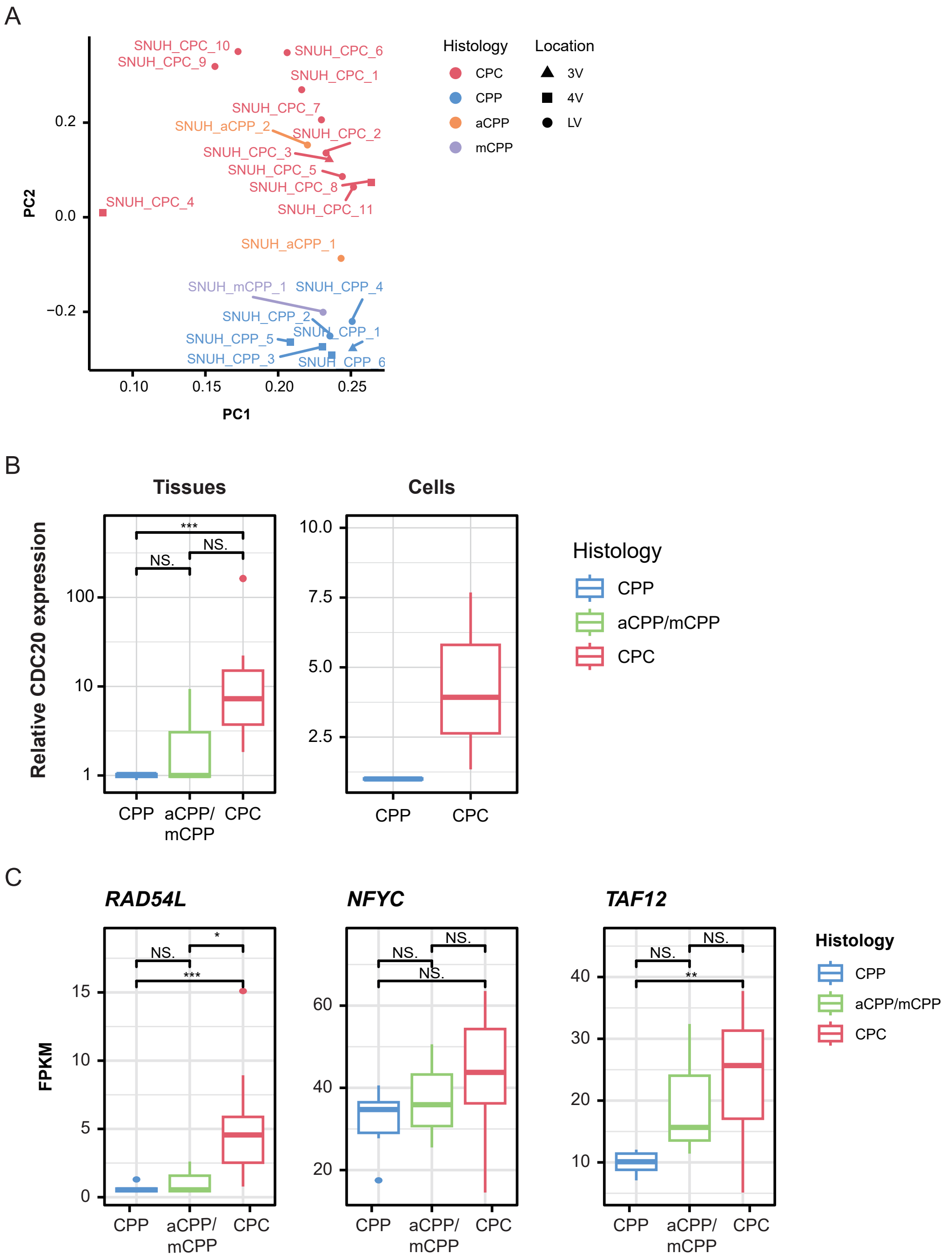

Supplementary Figure 4

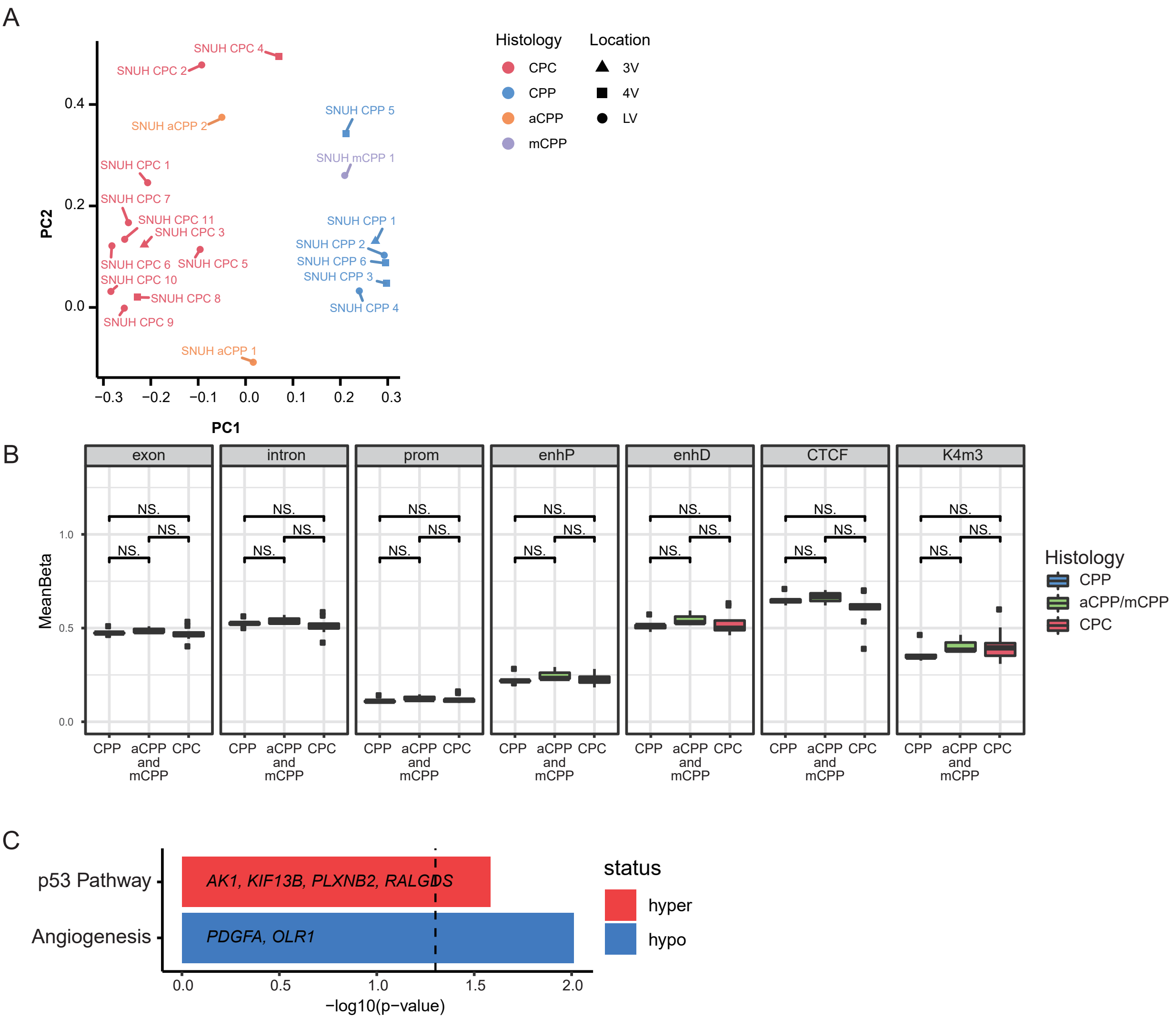

Supplement: Supplementary file 2 — Supplementary Material 2 [file 40478_2024_1814_MOESM2_ESM.pdf]
