# Supplementary material for: Comprehensive multiomics analysis reveals distinct differences between pediatric choroid plexus papilloma and carcinoma
Source: Acta Neuropathol Commun. 2024 Jun 12;12:93. doi: 10.1186/s40478-024-01814-y (PMC11167863; doi:10.1186/s40478-024-01814-y)
Supplement: Supplementary file 3 — Supplementary Material 3 [file 40478_2024_1814_MOESM3_ESM.docx]

**Supplementary Figure legends**

**Supplementary Figure 1.** SCNA profile of the CPT cohort. a. Summary of the SCNA status of each patient. b. Shared or group-specific arm-level SCNAs between CPP and CPC. c. Focal SCNAs with cytoband annotation. Significant focal SCNAs are represented in red and blue peaks, and cytoband labels for the common focal events between CPP and CPC are colored in red and blue, respectively, for gains and losses.

**Supplementary Figure 2.** Boxplot showing the number of predicted clonal groups between CPC and other subtypes. Significant differences between groups were calculated by t test.

**Supplementary Figure 3.** Validation of DEGs and previously reported genes. a. PCA using DEGs between CPP and CPC. b. Experimental validation of *CDC20* expression in tumor tissues and cultured cells via RT-qPCR. c. Boxplot illustrating the expression of three genes (*RAD54L*, *NFYC*, and *TAF12*) previously reported in Tong et al. [57] across different subtypes of CPT. Significant differences between groups are indicated by asterisks.: **P* < 0.05, ***P* < 0.01, ****P* <0.001 as calculated by the Wilcoxon rank-sum test.

**Supplementary Figure 4.** Methylation analysis of the CPT cohort. a. PCA using DMSs between CPP and CPC. b. Boxplot illustrating the degree of methylation in various genomic regions, grouped by CPT subtype. Significant differences between groups are indicated by asterisks.: **P* < 0.05, ***P* < 0.01 as calculated by *t* test. c. Enrichment test of genes with significant DMSs in the promoter region.
